# Diagnostic efficacy of the optical flow ratio in patients with coronary heart disease: A meta-analysis

**DOI:** 10.1371/journal.pone.0285508

**Published:** 2023-05-10

**Authors:** Huaigang Chen, Bin Li, Yanyan Xiao, Hong Wang, Maobin Kuang, Hanjin Sun, Liu Yang

**Affiliations:** 1 Medical College of Nanchang University, Nanchang, Jiangxi Province, China; 2 Department of Cardiology, Jiangxi Provincial People’s Hospital, The First Affiliated Hospital of Nanchang Medical College, Nanchang, Jiangxi Province, China; 3 Postgraduate School of Jiangxi University of Traditional Chinese Medicine, Jiangxi University of Traditional Chinese Medicine, Nanchang, Jiangxi Province, China; Baylor Scott and White, Texas A&M College of Medicine, UNITED STATES

## Abstract

**Background:**

Coronary atherosclerotic heart disease (CAD) remains one of the most serious diseases threatening human health and life. PCI (Percutaneous Coronary Intervention) is the most common treatment for patients with CAD. A rigorous and comprehensive assessment of coronary artery lesions is now needed before PCI, however, there is no consensus on how best evaluate the combination of various intracavitary imaging techniques. By merging the benefits of physiological assessment and high-definition imaging, the optical flow ratio (OFR) has emerged as a novel technology with promising prospects for application.

**Methods:**

A systematic review of the literature was conducted. Studies that met the criteria of the meta-analysis were considered to assess OFR and FFR (fractional flow reserve). And the summary values of sensitivity and specificity of diagnostic tests and summary receiver operating curves (SROC) were calculated.

**Results:**

A total of 5 studies were included. The sensitivity and specificity of OFR in the diagnosis of coronary artery lesions were 0.83 (95% CI: 0.75–0.88) and 0.94 (95% CI: 0.91–0.96), respectively; the positive likelihood ratio and the negative likelihood ratio were 14 (95% CI: 9.3, 21.3) and 0.18 (95% CI:0.13, 0.27), respectively. OFR showed good correlation and consistency with FFR.

**Conclusion:**

The new OFR technique achieve an encouraging diagnostic performance, which also showed good correlation and consistency with FFR.

## 1. Introduction

Coronary atherosclerotic heart disease (CAD) is still an important disease that endangers human health [1]. One of the most effective treatments is percutaneous coronary intervention (PCI). A rigorous and comprehensive assessment of coronary artery lesions is now needed before PCI. These assessments can be divided into the following two aspects: the morphological assessment of the diseased segment and the physiological assessment of myocardial ischemia. Currently, the commonly used assessment tools mainly include intravascular ultrasound (IVUS), optical coherence tomography (OCT), near-infrared spectroscopy (NIRS), and fractional flow reserve (FFR), etc. Each imaging technique has unique advantages and disadvantages [2]. Therefore, it is very important to select the most appropriate application scenario. For example, OCT provide a higher resolution. In the meantime, OCT requires an additional step of flushing the vessel with a contrast agent. Therefore, OCT is not suitable for coronary dissection. However, the automatic lumen calculation of OCT will provide more accurate data, while the calculation of the lumen area of IVUS requires manual correction. IVUS-guided stent implantation can significantly reduce the application of contrast agents, which is beneficial to patients with poor renal function [3]. Although IVUS has stronger lesion penetrability than OCT, it cannot penetrate heavily calcified plaques in the assessment of coronary calcification. In addition, fractional flow reserve (FFR) is the physiological “gold standard” for assessing the severity of coronary heart disease [4]. However, the practical application of FFR in the real world is very inadequate, mainly due to its invasive operation, high economic cost, and the need to use extra drugs, etc. Therefore, to date, there is no consensus on how best evaluate the combination of various intracavitary imaging tools. Obviously, to achieve the best assessment of the morphological and physiological, it is necessary to perform at least two items imaging examinations at the same time. The optical flow ratio (OFR), a new technique for FFR calculation based on OCT images, which combines physiological assessment with high-resolution imaging have been developed [5]. At present, this technique can accomplish FFR ultra-fast calculation, AI plaque automatic property recognition and coronary three-dimensional reconstruction. In recent years, some studies on the diagnostic efficacy of OFR in coronary artery lesions have been reported [6–10]. What is not yet clear is the diagnostic efficacy of OFR in patient with CAD. Therefore, for the first time, the aim of this meta-analysis was to evaluate the diagnostic efficacy optical flow ratio in patients with coronary heart disease.

## 2. Materials and methods

### 2.1 Search strategy

Two researchers, Huaigang Chen and Liu Yang, searched PubMed, EMBASE, clinical controlled trial database of the Cochrane Library and Sino-med database. The search period was from the establishment of the database to June 2022. The keywords are as follows: OFR, optical flow ratio, OCT or optical coherence tomography, OCT-based computed FFR, OCT-based computed fractional flow reserve, FFR-OCT. The S1 File provides a more comprehensive search formula for this study. It adheres to the guidelines outlined in the Preferred Reporting Items for Systematic Reviews and Meta-Analyses (PRISMA) statement [11].

### 2.2 Inclusion and exclusion criteria for the literature

Literature inclusion criteria were as follows: (1) study type: randomized controlled study, cohort study, and case‒control study; (2) the diagnostic efficacy in the study was evaluated by using FFR as the reference standard; (3) sufficient data were provided to calculate and construct a 2×2 contingency table that reports the number of true positives, false positives, false negatives, and true negatives.

The exclusion criteria were as follows:(1) repeated published literature; (2) only abstracts or conference compilations (incomplete information provided); (3) Literature reviews, expert comments, animal experiments or basic experiments, etc.

### 2.3 Literature screening

Two researchers, Huaigang Chen and Liu Yang, independently extracted data for each article. If no agreement can be reached, another author will mediate.

### 2.4 Data extraction and quality assessment

The following information was collected from the included studies: first author, year of publication, study type, inclusion and exclusion criteria, cut off value of OFR and FFR, general demographic characteristics, lesion characteristics, and diagnostic parameters. The risk of bias was assessed using Review Manager 5.4 and the Diagnostic Accuracy Study Quality Assessment Table 2 (QUADAS-2) [12]. It includes four parts: patient selection, index test, reference standard, process and timing. And a total of 11 question items (yes or no) was provided to help determine the risk of bias.

### 2.5 Statistical analysis

Stata 16.0 software was used to process the data, combine the sensitivity, specificity, and likelihood ratio results. At the same moment, plot forest plot, funnel plot, SROC plot, and predicted posttest probability plot were also plotted. Synthesizing data was achieved by a bivariate mixed-effects regression model. Among these, the funnel chart (Deeks’ method) is used to evaluate potential publication bias. The assessment of heterogeneity requires a preliminary evaluation of the attributes of both the population under examination (e.g. gender, pre-existing medical conditions) and the affected blood vessels. Such comparison may reveal differences between studies. Additionally, the Cochran’s Q test and I2 are employed. When p < 0.1 or I2 > 50%, there is significant heterogeneity between the studies.

## 3. Results

### 3.1 Characteristics of the included literature

The proposed search terms were searched in each database, 4192 relevant articles were first identified, which publication year was from January 1993 to July 2022.

**Fig 1:**
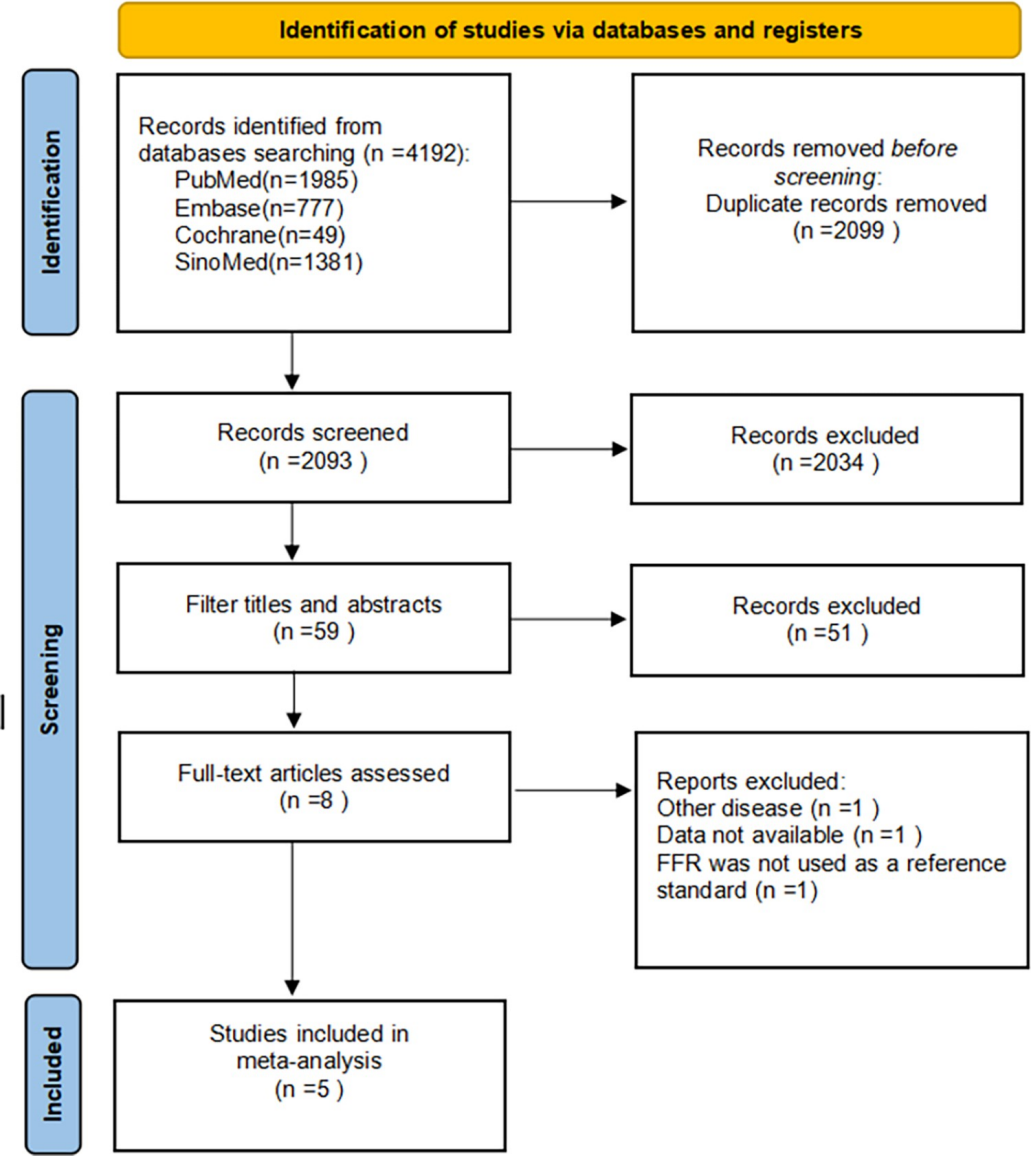
Flow chart for study screening.

After reading the title and abstract of the article, 51 articles that were not related to the purpose of the study were excluded. After further full-text reading, 3 articles were excluded, and 5 articles were ultimately included in this study. The flowchart is shown in Fig 1.

The detailed information of the five studies is described in Table 1, including the first author, the year of publication, the number of patients, the number of lesions, the study type, the FFR cutoff value, and the OFR cutoff value. In these studies, only one was a prospective study, and the remaining four were retrospective studies. Overall, these studies contained 545 patients and 599 vascular lesions. Table 2 shows the baseline level and lesion characteristics of the patients. Among them, 70.5% of the patients had hypertension, 63.5% had hyperlipidemia, and 379 (63.2%) of the patients had left anterior descending artery (LAD) lesions.

**Table 1:** Characteristics of included studies.

Studies	Year	No. of patients	No. of lesions	Type	FFR cutoff	OFR cutoff
Jiayue Huang	2020	181	212	Single-center, retrospective	0.80	0.80
Juan Luis	2020	59	75	Multicenter, prospective	0.80	0.80
Wei Yu	2019	118	125	Multicenter, retrospective	0.80	0.80
Jinyong Ha	2016	92	92	Single-center, retrospective	0.80	0.80
Sun-Joo Jang	2017	95	95	Single-center, retrospective	0.80	0.80

FFR = fractional flow reserve

OFR = optical flow ratio

**Table 2:** Baseline patient characteristics and Lesion location.

Studies	Jiayue Huang	Juan Luis	Wei Yu	Jinyong Ha	Sun-Joo Jang	Total
**Age (y)**	70	63.4±10.1	64.4±10.2	62.7±9.6	62.3±8.6	65.5
**Woman**	44(24.3%)	9(15.3%)	35(29.7%)	34(37%)	NR	122(27.1%) *
**BMI (Kg/m2)**	24.2±3.6	28.1	28.3±6.7	NR	24.6±2.6	25.9*
**Diabetes**	77(42.5%)	23(39%)	35(29.7%)	20(21.7%)	30(32%)	185(33.9%)
**Hypertension**	149(82.3%)	40(67.8%)	101(85.6%)	54(58.7%)	40(42%)	384(70.5%)
**Hyperlipidaemia**	133(73.5%)	31(52.5%)	78(66.1%)	64(69.6%)	40(42%)	346(63.5%)
**Smoker**	36(19.9%)	17(28.8%)	39(33.1%)	12(13%)	27(28%)	131(24%)
**Family history of CAD**	40(22.1%)	6(10.2%)	NR	NR	4(4%)	50(18.4%) *
**Pervious PCI**	118(65.2%)	40(60.7%)	26(22%)	NR	11(12%)	195(43%) *
**Pervious MI**	81(44.8%)	28(47.5%)	27(22.9%)	NR	NR	136(38%) *
**LAD**	128(60.4%)	39(52%)	77(61.6%)	92(100%)	43(45%)	379(63.2%)
**LCX**	36(17%)	4(5.3%)	17(13.6%)	0	9(10%)	66(11%)
**RCA**	46(21.7%)	23(30.7%)	30(24%)	0	43(45%)	142(23.7%)

BMI = body mass index

CAD = coronary atherosclerotic disease

LAD = left anterior descending

PCI = percutaneous coronary intervention

MI = myocardial infarction

LCX = left circumflex

RCA = right coronary artery

NR = not reported

* The data that represents the sample was obtained by excluding the NR values

### 3.2 Quality assessment and publication bias

**Fig 2:**
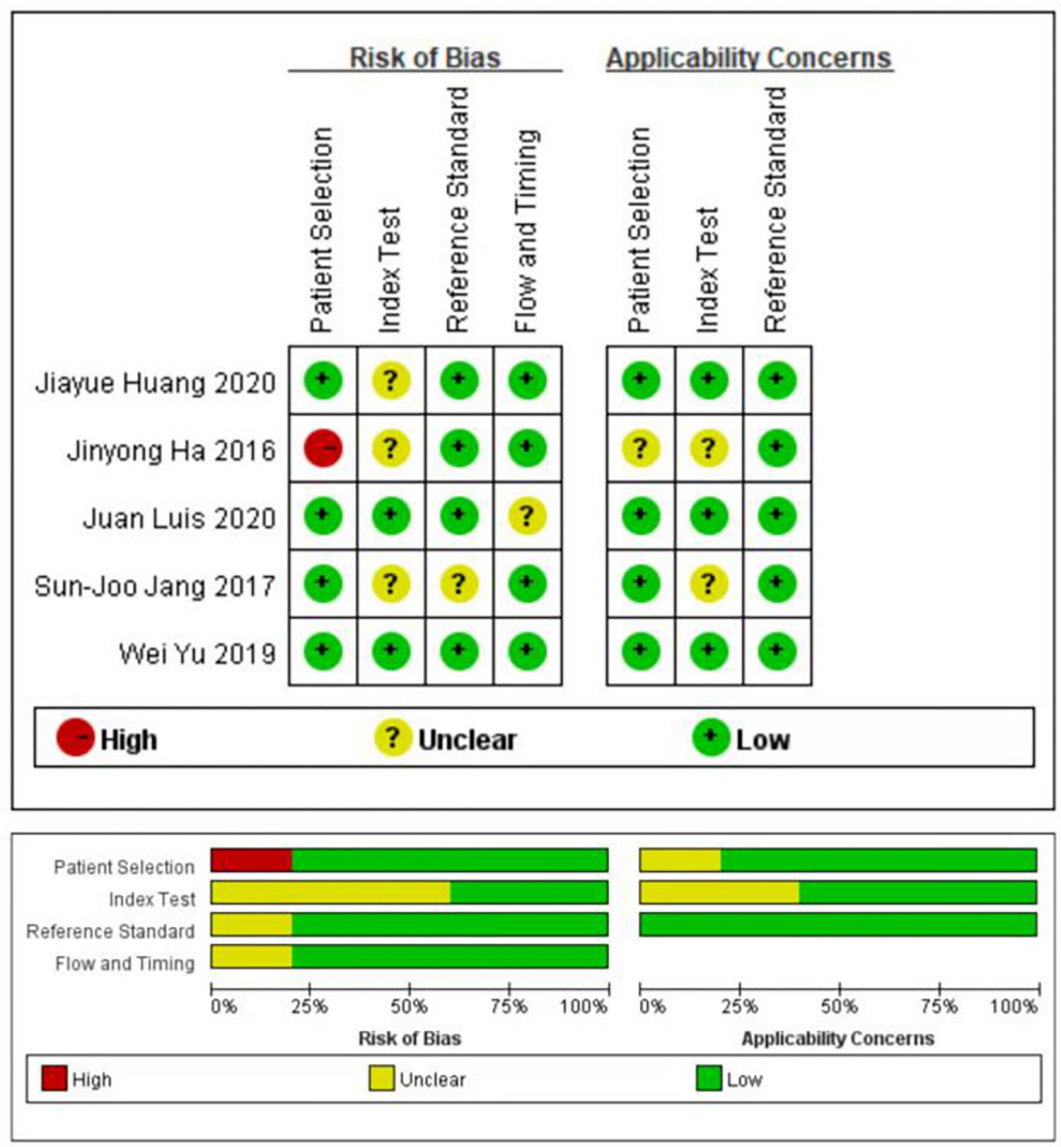
Methodological quality graph and summary.

**Fig 3:**
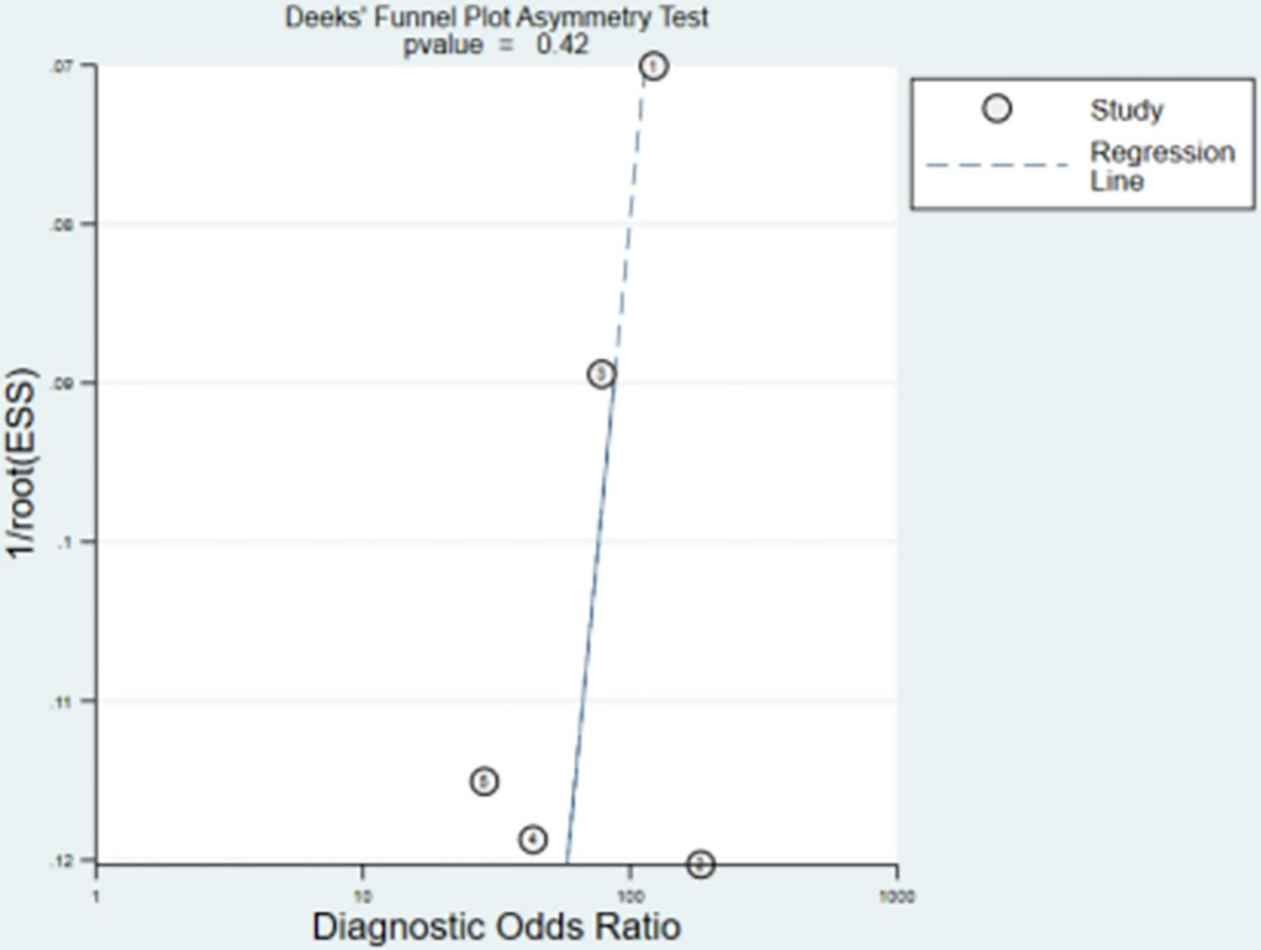
Deeks’ funnel plot asymmetry test.

The methodological quality of the OFR study is summarized in Fig 2. The overall quality of the study ranged from moderate to high. The five studies underwent statistical pooling included one study with low risk of bias and one study with high risk of bias. According to the Deeks’ funnel plot (Fig 3), the p value is 0.42, suggesting that the possibility of publication bias is small.

### 3.3 Diagnostic efficacy of OFR in patient with coronary artery disease

**Fig 4:**
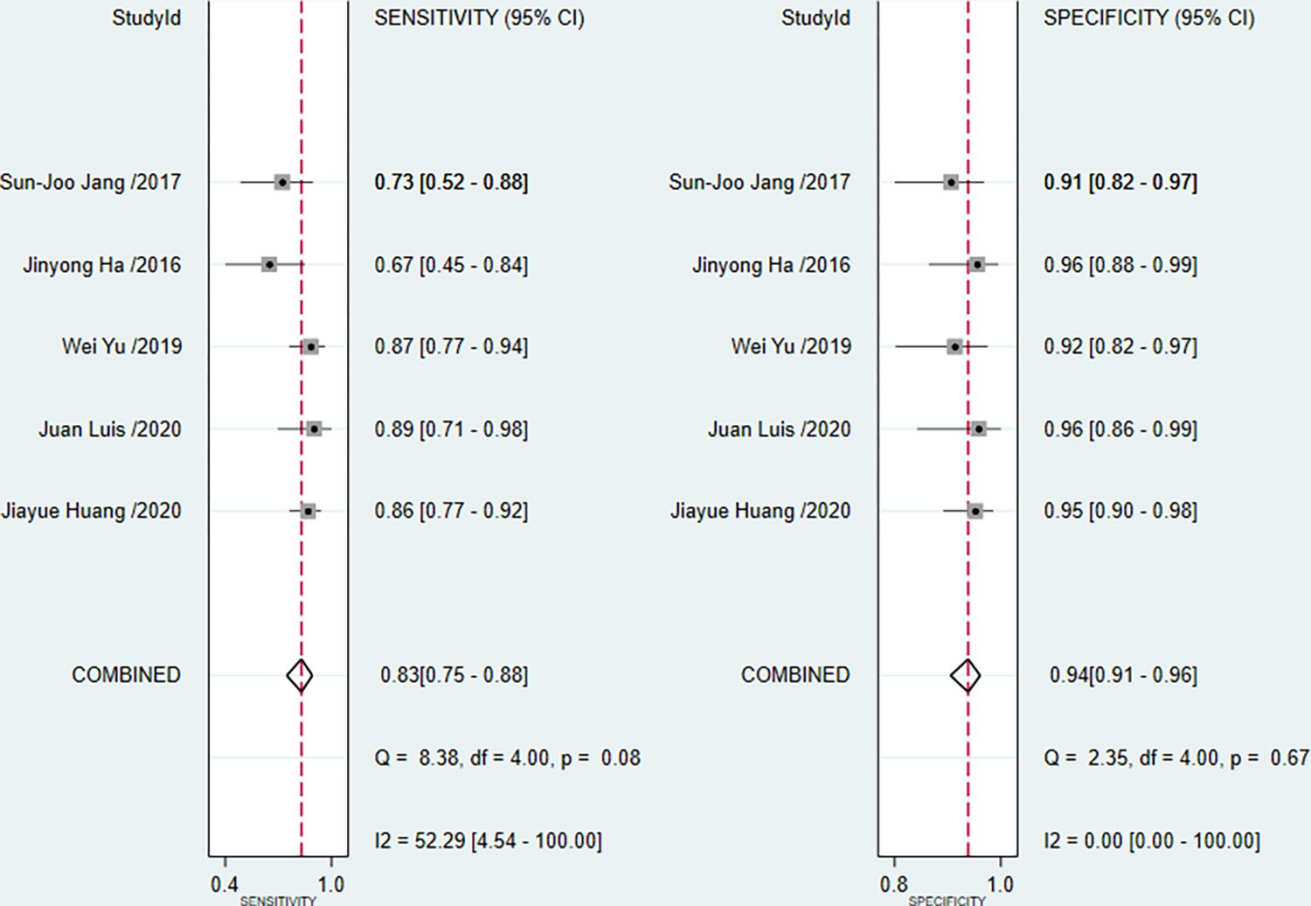
Forest plot of pooled estimates for diagnostic test data.

**Fig 5:**
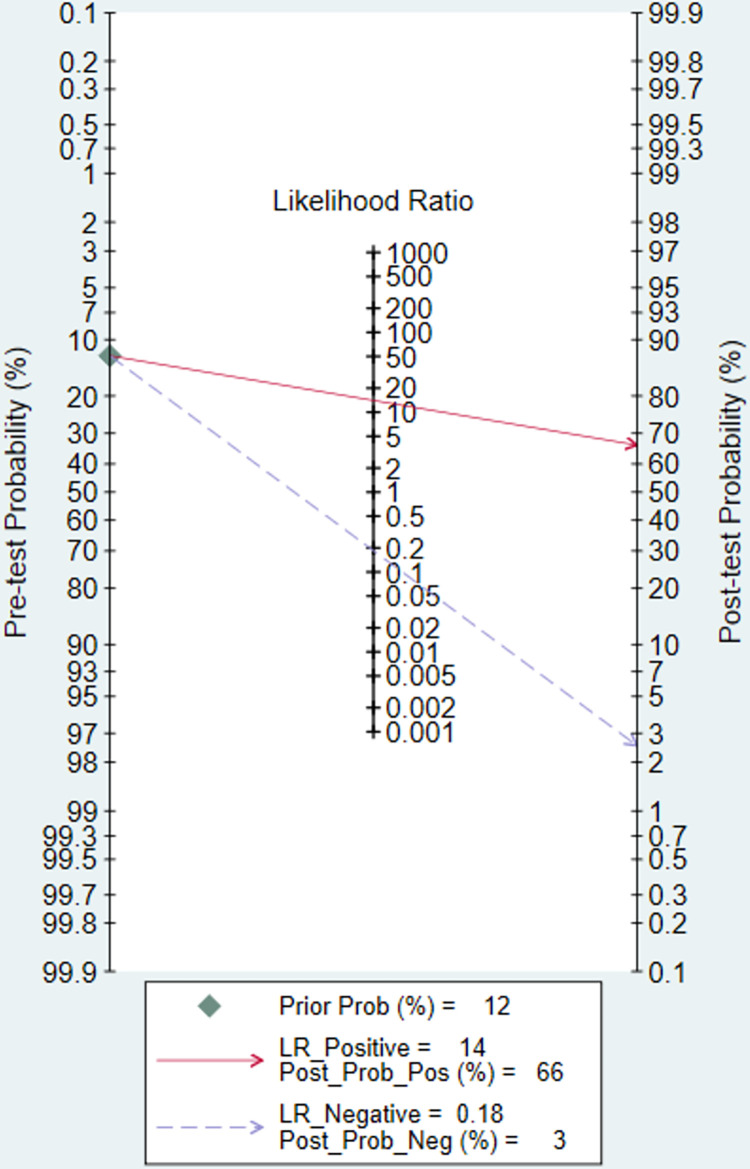
Predicted posttest probability plot.

**Fig 6:**
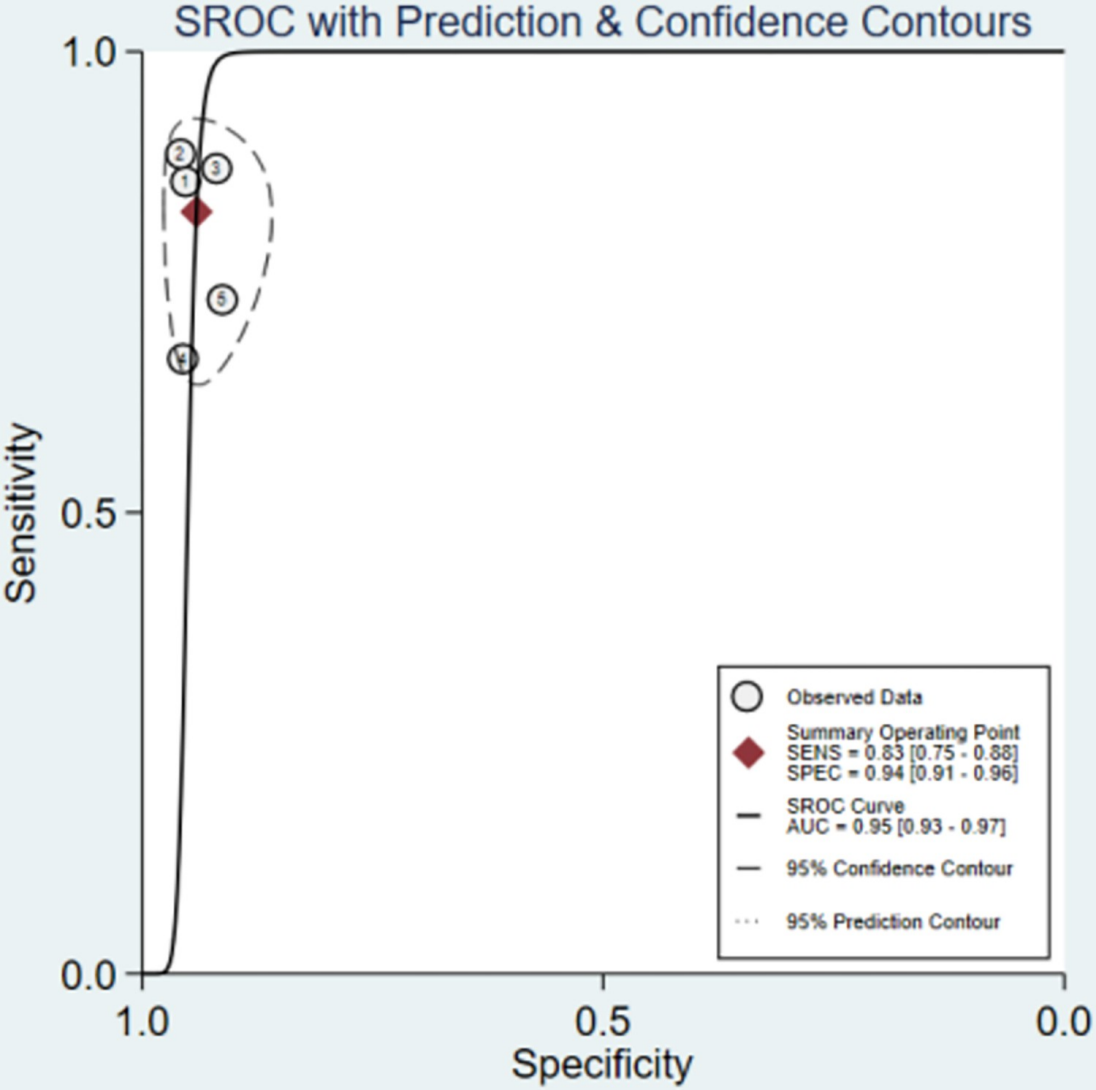
Summary receiver operator curve (SROC).

In the five included studies, the accuracies of OFR in the diagnosis of coronary artery lesions were above 85% (Table 3). However, the sensitivity of OFR was different in these studies (the highest value was 92%, and the lowest value was 68.7%). The results showed that the sensitivity of the OFR in the diagnosis of coronary heart disease was 0.83 (95% confidence interval CI: 0.75–0.88), which shown in the forest plot (Fig 4). However, there was heterogeneity between the studies (I^2^>50%, p<0.1). After pooling, right side of Fig 4 shows that the specificity of the 5 studies were 0.94 (95% CI: 0.91–0.96). The posttest probability plot (Fig 5) shows that the estimated values of LR+ and LR- of OFR are 14 (95%CI: 9.3–21.3) and 0.18(95% CI: 0.13–0.27), respectively. Moreover, when the prevalence of coronary heart disease is about 12% [13, 14], OFR can increase the positive diagnosis rate to 66% (and the negative diagnosis rate was 3%) at this point, which has a high diagnostic value. The summary receiver operator curve (SROC) was shown in Fig 6. The area under the curve (AUC) was 0.95(95%CI: 0.93–0.97). These results suggest that OFR has excellent diagnostic efficacy for coronary artery stenosis.

**Table 3:** Diagnostic performance of OFR.

Studies	OFR≤0.80							
	Accuracy	Sensitivity	Specificity	PPV	NPV	LR(+)	LR(—)	AUC
Jiayue Huang	92% (88–95)	86% (77–93)	95% (90–98)	92% (84–97)	91% (85–95)	18.2(8.3–39.9)	0.2(0.1–0.3)	0.97(0.93–0.99)
Juan Luis	93% (86–99)	92% (73–99)	93% (82–99)	88% (69–98)	96% (85–99)	13.8(4.6–41.3)	0.1(0.0–0.3)	0.95(0.86–0.99)
Wei Yu	90% (84–95)	87% (77–94)	92% (82–97)	92% (82–97)	88% (77–95)	10.8(4.6–25.2)	0.1(0.1–0.3)	0.93(0.87–0.97)
Jinyong Ha	88%	68.70%	95.60%	84.20%	89%	NR	NR	NR
Sun-Joo Jang	86%	73% (52–88)	91% (81–96)	76% (55–90)	90% (80–96)	NR	NR	NR

OFR = optical flow ratio; PPV = positive predictive value

NPV = negative predictive value

LR = likelihood ratio

AUC = area under the curve

## 4. Discussion

As mentioned in the background, this is the first meta-analysis that compares the diagnostic efficacy of the two assessment techniques (OFR and FFR). In this study, the new OFR technique achieve an encouraging diagnostic performance. It was found that when FFR was used as reference, OFR and FFR have high consistency in the diagnosis of coronary stenosis. The sensitivity and specificity of OFR in the diagnosis of coronary stenosis were 83% and 94%, respectively. OFR has good diagnostic performance in the assessment of coronary stenosis.

Among the five included studies, Jinyong Ha et al. reported the OFR technique for the first time. In the meantime, the results showed that OCT-FFR had a major role in detecting functionally significant ischemic coronary artery [6]. His study mainly included patients with left anterior descending artery lesions. He and his coworkers found that OFR was highly specific in identifying functional ischemia in left anterior descending artery lesions (95.6%). However, the OCT-FFR technique used by Jinyong Ha ignores the collateral branches to simplify the solution of the Navier‒Stokes equation for the intracoronary shear stress. In contrast, a study by Yingguang Li et al. on the effect of coronary side branch modeling which explains the relationship between coronary side branches and shear stress at the lesion further [15]. They compared the traditional single-conduit model (SCM) with the true anatomical tree model (TM) that includes collateral branches and found that the side was ignored when simulating coronary artery reconstruction. Under these conditions, this would underestimate the distal coronary pressure and aortic pressure (Pd/Pa), which ultimately resulting in an inaccurate estimate of shear stress (SS) in the stenotic region. This may lead to a decrease in diagnostic accuracy, which is similar to that which we report here. This also suggests that it deserves to investigate if a simplified version OCT-FFR will provide better diagnostic performance. For the first time, Jiayue Huang et al. compared quantitative flow ratio (QFR) with OFR [7]. Finally, the results showed that the consistency between OFR and FFR was significantly better than that of QFR (92% vs. 87%). This can occur especially in patients with history of PCI or myocardial infarction, which agreed with our study. This might be because in patients with complex coronary lesions, interference from overlapping vessels or calcification may lead to poor quality angiographic images, which resulting in inaccurate or incomplete QFR calculations. On the other hand, Yuming Huang et al performed a direct comparison of OFR and QFR in diagnosing a single vascular stenosis and found a significant correlation between the two (r = 0.86, p< 0.001) [16]. In the study of Wei et al., the advantage (OFR) in bifurcation lesions was emphasized [8]. In this population, the accuracy of the minimum lumen area (MLA) calculated by OCT for the diagnosis of ischemia (FFR<0.8) was only 74%. This result is not difficult to explain because the blood flow reserve at the lesion is determined by the characteristics of lumen area, lesion length and surface roughness. Obviously, MLA is just one of the indicators [17]. However, the area under the curve (AUC) for the diagnosis of ischemia can be increased from 0.80 to 0.93 by adding the OFR evaluation tool. These results further support the idea of our conclusion. The reason why is that OFR can accurately calculates the lumen size of the coronary bifurcation [18, 19]. The study by Juan Luis et al. is the first prospective study to demonstrate the feasibility of OFR [9]. Moreover, this study demonstrates that the speed of retraction has a negligible impact on the results during OFR operations. As long as the same coronary artery segment is selected, the diagnostic accuracy remains almost constant regardless of the retracement speed (But there is no denying that a high retraction speed of 36mm/S is preferred). It could be argued that the positive results were due to the longer retracement, which obtain images of longer coronary artery segments. That means that OFR is less affected by subjective factors. This finding broadly supports our results. Sun-Joo Jang et al. also compared the differences between the FFR-OCT_AFD_ and FFR-OCT_CFD_ models in the diagnosis efficacy of coronary artery lesions, and the results showed that both of them had good consistency with FFR [10]. The accuracy of the former is 86%, and the specificity of the FFR-OCT_AFD_ is slightly higher than that of the latter (94% (95%CI:85%-98%) vs. 91% (95%CI:81%-96%). This may be due to the fact that the FFR-OCT_AFD_ are calculated based on Poiseuill’s and Ohm’S law used.

The morphology of coronary plaques has a potential impact on the accuracy of optical flow ratio (OFR). A study analyzed 230 coronary arteries with moderate stenosis among 198 patients in a retrospective manner [20]. The classification of diseased vessels was based on the morphology of coronary artery plaque, which included thin cap fibrous atherosclerotic plaque (TCFA), lipid plaque volume (LPV), fibrous plaque volume (FPV), and calcified plaque volume (CPV). These vessels were categorized into three subgroups. According to research, the precision of OFR calculations is impacted by lipid atherosclerotic plaques, as opposed to calcified or fibrotic plaques. One possible explanation is that lipid-rich plaques are more flexible and capable of greater lumen expansion and strain than rigid fibers or calcified plaques. This flexibility may result in an underestimation of wall rigidity in OFR calculations, ultimately compromising the accuracy of the final results [21].

In terms of research content, some limitations need to be acknowledged. Firstly, we found that there were few studies related to OFR, and only 5 studies with 545 patients were included. Secondly, only one of the included studies was a prospective study, which may lead to an overestimation of the diagnostic accuracy of OFR. In the future, this conclusion needs to be further confirmed by further studies.

## 5. Conclusion

The sensitivity and specificity of OFR in diagnosing coronary stenosis lesions reached 83% and 94%, respectively, which was in good agreement with FFR. These findings might help develop appropriate interventional strategies for interventional physicians and radiologists.

## Supporting information

S1 File Detailed strategy for literature search. (DOCX)

S2 File Detailed data of the study. (XLSX)
