# Supplementary material for: Diagnostic efficacy of the optical flow ratio in patients with coronary heart disease: A meta-analysis
Source: PLoS One. 2023 May 10;18(5):e0285508. doi: 10.1371/journal.pone.0285508 (PMC10171614; doi:10.1371/journal.pone.0285508)
Supplement: S1 File — (DOCX) [file pone.0285508.s001.docx]

**Search formulas**

optical flow ratio[Title/Abstract]

(optical flow ratio[Title/Abstract]) AND (OCT[Title/Abstract])

((optical flow ratio[Title/Abstract]) ) AND (fractional flow reserve[Title/Abstract])

(((optical flow ratio[Title/Abstract]) ) ) AND (FFR[Title/Abstract])

(OFR[Title/Abstract]) OR (ofr[Title/Abstract])

((OFR[Title/Abstract]) ) AND (OCT[Title/Abstract])

(((OFR[Title/Abstract]) ) ) AND (optical coherence tomography[Title/Abstract])

OCT-based computed FFR[Title/Abstract]

OCT-based computed fractional flow reserve[Title/Abstract]

OCT-FFR[Title/Abstract]
